# Comparison of pressure controlled, volume controlled, and volume guaranteed pressure controlled modes in prone position in patients operated for lumbar disc herniation: A randomized trial

**DOI:** 10.1097/MD.0000000000037227

**Published:** 2024-02-09

**Authors:** Ferim Sakize Gunenc, İlkana Seyidova, Sule Ozbilgin, Koray Ur, Volkan Hanci

**Affiliations:** a Dokuz Eylul University, School of Medicine, Department of Anesthesiology and Intensive Care, Izmir, Turkey; b Dokuz Eylul University, School of Medicine, Department of Neurosurgery, Izmir, Turkey.

**Keywords:** prone position, spine surgery, ventilation modes

## Abstract

**Background:**

To compare pressure-controlled ventilation (PCV), volume-controlled ventilation (VCV), and pressure-controlled ventilation-volume guaranteed (PCV-VG) modes in patients undergoing spinal surgery in the prone position under general anesthesia.

**Methods:**

The study included 78 patients aged 20 to 80 years, American Society of Anesthesiologists 1–2, scheduled for lumbar spinal surgery. Patients included in the study were randomly divided into 3 groups Group-VCV; Group-PCV; Group-PCV-VG. Standard anesthesia protocol was applied. In addition to routine monitoring, train of four and BIS monitoring were performed. All ventilation modes were set with a target tidal volume of 6 to 8 mL/kg, FiO2: 0.40–0.45 and a respiratory rate of normocarbia. Positive end-expiratory pressure: 5 cm H_2_O, inspiration/expiration ratio = 1:2, and the maximum airway pressure:40 cm H_2_O. Hemodynamic, respiratory variables and arterial blood gases was measured, 15 minutes after induction of anesthesia in the supine position (T1), after prone position 15 minutes (T2), 30 minutes (T3), 45 minutes (T4), 60 minutes (T5), 75 minutes (T6), 90 minutes (T7).

**Results:**

There was no significant difference between the groups in patient characteristics. SAP, DAP, mean arterial pressure, and heart rate decreased after being placed in the prone position in all groups. Hemodynamic variables did not differ significantly between the groups. partial arterial oxygen pressure and arterial oxygen saturation levels in blood gas were found to be significantly higher in Group-PCV-VG compared to Group-PCV and Group-VCV in both the supine and prone positions. Ppeak and plateau airway pressure (Pplato) values increased and dynamic lung compliance (Cdyn) values decreased after placing the patients in the prone position in all groups. Lower Ppeak and Pplato values and higher Cdyn values were observed in both the supine and prone positions in the Group-PCV-VG group compared to the Group-PCV and Group-VCV groups.

**Conclusion:**

PCV-VG provides lower Ppeak and Pplato values, as well as better Cdyn, oxygenation values compared to PCV and VCV. So that PCV-VG may be an effective alternative mode of mechanical ventilation for patients in the prone position during lumbar spine surgery.

## 1. Introduction

During the operation, the position of the patients and the ventilation management applied may cause some changes in vital parameters. The aim of ideal ventilation is to protect the lungs and provide acceptable gas changes. For this purpose, minute ventilation should be performed without causing any increase in pressure and volume, while at the same time avoiding low expiratory lung volumes. When performing mechanical ventilation, it is important to prevent lung injury caused by barotrauma, volutrauma, atelectrauma, and biotrauma. Changes in functional residual capacity and closure volumes in the supine position and under anesthesia may lead to ventilation-perfusion inequalities and hypoxemia.^[1]^

Prone position is necessary to allow surgical access during posterior spine surgery.^[2]^ When the patient is in the pronated position, the increased intrathoracic pressure leads to a decrease in venous return and left ventricular compliance, hence the cardiac index. Pulmonary physiology is also affected in the prone position.^[3]^

When an anesthetized patient is rotated to the prone position, ventilation/perfusion ratio (V/Q), compliance and dynamic lung compliance (Cdyn) decrease and peak airway pressure (Ppeak) increases to achieve the set tidal volume (VT) because free abdominal and chest movements are not achieved.^[2,3]^

The abdominal wall, which holds the entire body weight in the prone position, causes difficulty in diaphragm movement and limitations in VT.^[2]^

When lung compliance is reduced, high airway pressure is required to provide adequate ventilation to the patient. This high airway pressure can reduce venous return to the heart and increase systemic venous pressure, including the epidural vein, as the epidural veins are connected to the inferior caval vein by a valveless venous system. This can lead to excessive bleeding, decreased spinal perfusion pressure and neurologic complications.^[3]^

The mechanical ventilation mode refers to the respiratory support method.^[4]^ Volume controlled ventilation (VCV) and pressure controlled ventilation (PCV) are the basic modes of controlled mechanical ventilation used during general anesthesia. Volume-guaranteed pressure-assisted and pressure-controlled modes (PCV-VG) are available on newer anesthesia machines. PCV-VG has the advantages of both VCV and PCV modes.^[5–7]^

Although there are previous studies comparing PCV-VG, VCV, and PCV in different surgeries,^[2,3,6,7]^ there are no studies comparing PCV-VG, VCV, and PCV in patients in the pron position.

The hypothesis of this study was that PCV-VG mode would provide adequate VT with lower peak airway pressures in patients undergoing spinal surgery in the prone position.

To test this hypothesis, the aim of this study was to determined and compare the effects of PCV, VCV, and pressure-controlled ventilation-volume guaranteed (PCV-VG) modes on hemodynamic and respiratory parameters and to determine the optimum ventilation mode for patients undergoing spinal surgery in the pron position under general anesthesia.

## 2. Material and Methods

This study was planned as a prospective and observational study. After obtaining the approval of the Non-Interventional Ethics Committee of Dokuz Eylul University Faculty of Medicine (Ethics Committee Decision No: 2022/08-05), patients who underwent lumbar spinal surgery under general anesthesia in the pronated position in the Central Operating Room of Dokuz Eylul University Hospital between October 2021 and October 2022 were included in the study. Written informed consent was obtained from all patients participating in the study.

The study included 78 patients aged 20 to 80 years with American Society of Anesthesiology (ASA) 1–3 who were scheduled for lumbar spinal surgery. After informed consent was obtained, the patients were randomly (using table of random numbers) divided into 3 groups according to the ventilation mode. Patients in Group-VCV were ventilated using volume controlled ventilation mode; patients in Group-PCV were ventilated using pressure controlled ventilation mode; patients in Group-PCV-VG were ventilated using the pressure-controlled ventilation-volume guaranteed mode.

**Figure 1.**
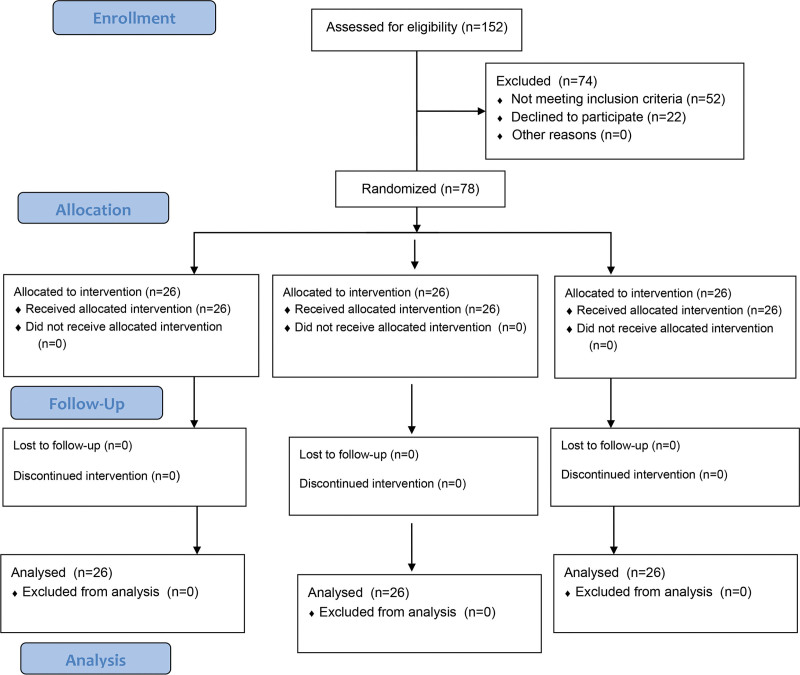
Flow diagram of the study.

Patients who did not want to participate in the study, patients with communication difficulties due to language problems and deafness, patients with a body mass index (BMI) ≥ 30 kg/m^2^, patients with comorbid diseases such as drug allergy, uncontrolled diabetes mellitus and hypertension, liver or kidney failure, psychiatric disorders, alcohol abuse, history of malignancy, severe lung disease were excluded (Fig. 1).

### 2.1. Method of anaesthesia

The anesthesia device used for mechanical ventilation (Drager, Zeus Infinity Empowered; Drager Medical AG & Co. KG, Germany) was automatically tested preoperatively and the suitability of the breathing circuit (Altech AL-1121; Altera Ltd., Izmir, Turkey and Zeus MK04248 reusable Anesthesia set) was determined by autotesting. Respiratory mechanics data such as Ppeak, plateau airway pressure (Pplato), VT, and Cdyn (dynamic compensation) were recorded by monitoring the anesthesia machine. All patients underwent standard anesthesia protocol and no additional drugs other than routine anesthetic drugs were used.

In addition to routine monitoring, train of four (TOF) and bispectral index (BIS) monitoring were performed. Quadruple stimulation (TOF) was used at the beginning of the operation to determine the optimal time for tracheal intubation, during the operation to monitor muscle blockade and at the end of the operation to monitor the return of muscle strength. After induction and before muscle relaxant administration, the ulnar nerve was stimulated with a 50 mA current in 15 seconds cycles with the TOF device and the contraction of the adductor pollicis muscle was controlled with quadruple stimulation. The patient was intubated when TOF rate = 0%. Patients were extubated when TOF was 90%. Adequate depth of anesthesia throughout the entire case was ensured by BIS monitoring, which is currently the most reliable method of depth of anesthesia measurement available. In the study, BIS values between 40 to 60 were used as a reference for adequate depth of anesthesia.

After induction of anesthesia, a cannula was placed in the radial artery for continuous arterial pressure monitoring and blood sampling. All ventilation modes were adjusted to maintain a target VT of 6 to 8 mL/kg, FiO2: 0.40–0.45 and respiratory rate normocarbia. End-expiratory positive pressure: 5 cm H_2_O, inspiratory/expiratory ratio = 1:2, maximum airway pressure at which the ventilator would alarm was programmed as 40 cm H_2_O.

Hemodynamic variables, respiratory variables and arterial blood gases were recorded 15 minutes after induction of anesthesia in supine position (T1), 15 minutes (T2), 30 minutes (T3), 45 minutes (T4), 60 minutes (T5), 75 minutes (T6), 90 minutes (T7) after being placed in pron position.

Respiratory mechanics parameters such as VT, Ppeak, Pplato, and mean airway pressure (Pmean), C-dyn, partial arterial oxygen pressure (PaO_2_) in arterial blood gas analysis, arterial carbon dioxide pressure (PaCO_2_), pH and oxygen saturation (arterial oxygen saturation [SaO_2_]), hemodynamic parameters heart rate (HR), systolic blood pressure, diastolic blood pressure (DBP), mean arterial pressure (MAP) were recorded at time points T1–T7.

### 2.2. Power analiz

The number of patients to be included in our study was determined with power analysis. For power analysis, https://clincalc.com/stats/samplesize.aspx website and power analysis program were used. The hypothesis of this study was that pressure controlled ventilation-volume guaranteed (PCV-VG) mode would provide adequate VT with lower peak airway pressures in patients undergoing spinal surgery in the prone position. In a study conducted by Yong SC et al,^[2]^ airway pressures in the prone position in patients ventilated with VCV were determined to be 15.56 ± 2.12. As determined by Yong SC et al^[2]^ airway pressures value (15.56 ± 2.12) with alpha error 0.05, 15% difference between the groups and 95% power, required a sample number of at least 21 individuals for the groups. 26 patients were included in the groups, with a 25% drop-out (patient loss) planned during follow-up.

### 2.3. Statistical analysis

We used the SPSS (Statistical Package for the Social Sciences) 24.0 package program to analyze the data of our research. We expressed frequent variables as number (n) and percentage (%). We used the Pearson chi-square test for group comparisons of frequency-indicating data. We examined the normal test assumptions of variables with continuous values using Kolmogorov, Smirnov, and Shapiro–Wilk tests. We expressed variables with continuous values whose distribution pattern conformed to normal distribution as mean ± standard deviation. We expressed variables with continuous values whose distribution pattern did not follow a normal distribution as median [interquartile range (IQR)]. We tested continuous value data using t test, One way ANOVA test, Mann–Whitney U test, Kruskal Wallis test, taking into account the number of groups and normality test results. We considered *P* values below .05 to indicate statistical significance.

## 3. Results

ASA I–II patients aged 18 to 65 years who were scheduled to undergo elective lumbar spinal surgery by the Department of Neurosurgery in the central operating room of Dokuz Eylül University Faculty of Medicine were included in the study. Seventy-eight patients from VCV, PCV, and PCV-VG groups were included in the study with 26 patients in each group. The study was completed in all patients (Fig. 1).

There was no statistically significant difference between the groups included in our study in terms of gender, ASA risk group distribution, mean age, mean height, mean weight and mean BMI (*P* > .05) (Table 1).

**Table 1:** Demographic data of the patients.

	GRUP-VCV n = 26	GRUP-PCV n = 26	GRUP- PCV-VG n = 26	*P*
Age (years)*	47.9 ± 13.9	47.5 ± 13.6	47.5 ± 12.5	.991†
Height (cm)**	168.00 (158.00–176.25)	170.00 (161.50–175.00)	165.00 (160.00–175.00)	.678††
Weight (kg)*	77.5 ± 11.2	78.6 ± 10.1	77.1 ± 9.1	.862†
BMI**	28.00 (26.32–29.22)	27.79 (25.95–29.25)	28.53 (26.67–29.53)	.761††
Gender (F/M)***	13 (50%)/13 (50%)	13 (50%)/13 (50%)	13 (50%)/13 (50%)	>.999†††
ASA I/II***	6 (23.1%)/20 (76.9%)	6 (23.1%)/20 (76.9%)	7 (26.9%)/19 (73.1%)	.933†††

ASA = The American Society of Anesthesiologists, BMI *=* body mass index, F = female, M = male.

* Mean±SD.

** Median (IQR).

*** n (%).

† Oneway ANOVA test.

†† Kruskal–Wallis test.

††

†Chi-Square test.

**Figure 2.**
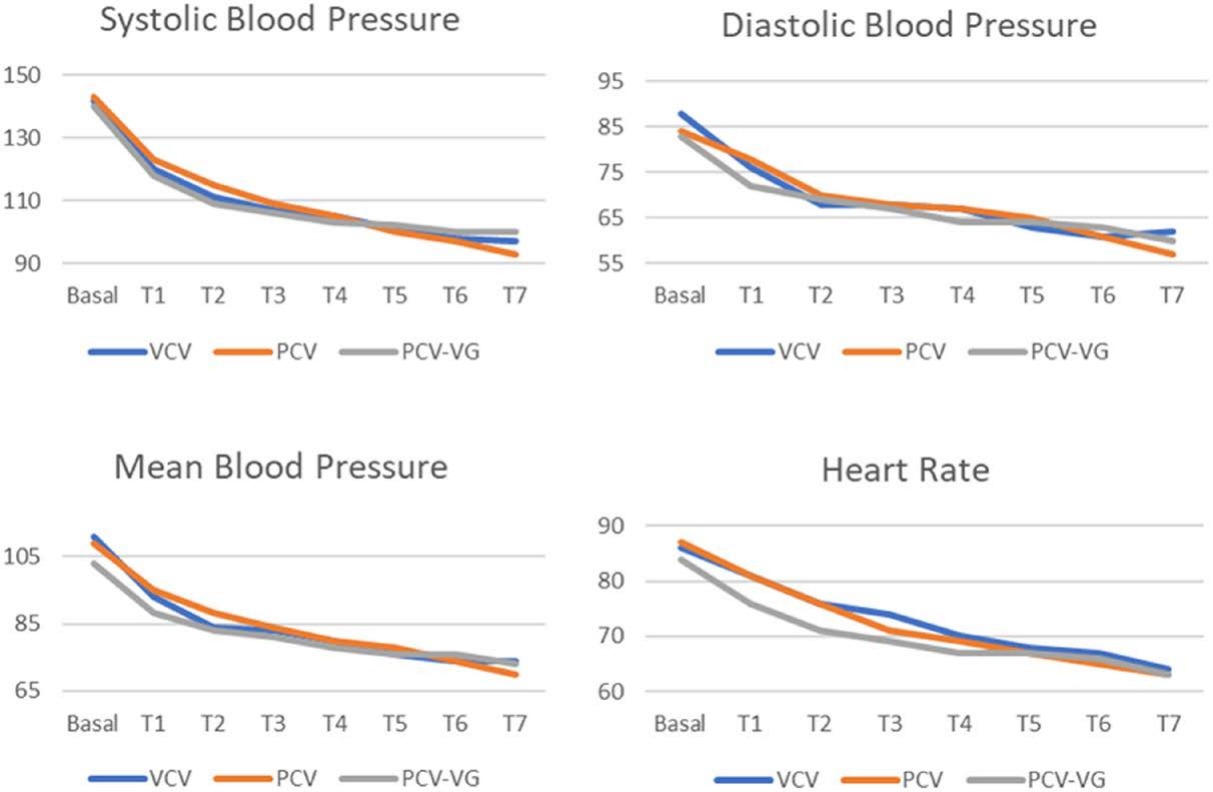
Blood pressure (mm Hg) and heart rate (beat/min) values in groups.

In all 3 groups, there were no significant differences between the groups in SDB, DBP, BP and HR in the pron and supine positions (*P* > .05) (Fig. 2).

**Figure 3.**
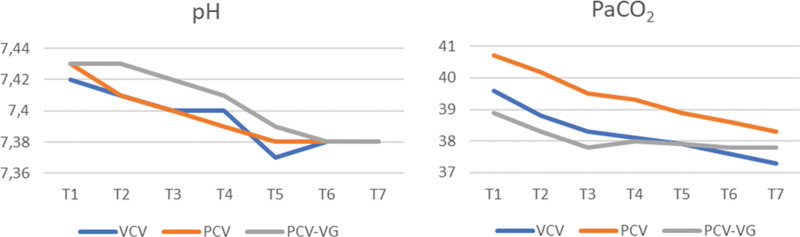
Arterial blood gas pH and PaCO_2_ (mm Hg) values in groups.

In all 3 groups, arterial blood gas pH and PaCO_2_ values obtained after position change did not differ between the groups (*P* > .05) (Fig. 3).

When the change of PaO_2_ and SaO_2_ averages according to time was evaluated in the groups, a statistical difference was found between the measurement points (*P* < .05). PaO_2_ and SaO_2_ values increased significantly in all 3 groups after changing from supine to pron position. PaO_2_ and SaO_2_ values were statistically higher in group PCV-VG compared to group PC and group VCV (*P* < .05). Oxygenation was determined to be better in group PCV-VG (Table 2).

**Table 2:** Arterial blood gas PaO_2_ (mm Hg) and SaO_2_ (%) values (mean ± SD).

PaO_2_	T1	T2	T3	T4	T5	T6	T7
VCV	158.1 ± 27.7	168.1 ± 27.7	169.9 ± 27.8	170.8 ± 27.8	170.9 ± 27.8	171.3 ± 27.7	167.1 ± 31.4
PCV	175.7 ± 44.8	191.2 ± 44.7	193.3 ± 44.6	194.7 ± 44.7	195.9 ± 44.5	197.0 ± 44.6	208.5 ± 36.4
PCV-VG	182.9 ± 28.4	208.5 ± 27.8	208.4 ± 25.2	209.1 ± 26.1	208.6 ± 27.7	209.8 ± 26.8	208.7 ± 27.4
*P* (PaO_2_)	**.034** *	**<.001** *	**<.001** *	**<.001** *	**.001** *	**<.001** *	**<.001** *
SaO_2_	**T1**	**T2**	**T3**	**T4**	**T5**	**T6**	**T7**
VCV	97.6 ± 0.5	98.1 ± 0.4	98.3 ± 0.3	98.4 ± 0.4	98.4 ± 0.4	98.5 ± 0.4	98.5 ± 0.4
PCV	98.1 ± 0.5	98.6 ± 0.4	98.8 ± 0.4	98.9 ± 0.4	99.0 ± 0.4	99.1 ± 0.3	99.1 ± 0.3
PCV-VG	98.7 ± 0.4	99.3 ± 0.3	99.4 ± 0.3	99.4 ± 0.3	99.5 ± 0.3	99.6 ± 0.2	99.7 ± 0.1
*P* (SaO_2_)	**<.001** *	**<.001** *	**<.001** *	**<.001** *	**<.001** *	**<.001** *	**<.001** *

Bold value indicates statistically significant.

* One-way ANOVA test.

Mean Ppeak and Pplato pressure levels increased significantly in all 3 groups after changing the position from supine to prone. During the study period, the PCV-VG group had lower Ppeak and Pplato values than the PCV and VCV group in both supine and prone positions. The highest Ppeak and Pplato values were observed in group VCV (*P* < .05). In all 3 groups, a significant decrease in dynamic compliance values was observed in the transition from supine to pronated position. Cdyn was higher in Group PCV-VG than in Group PCV and Group VCV in both supine and pron positions during the study period. The lowest Cdyn was observed in Group VCV (*P* < .05) (Table 3).

**Table 3:** Peak inspiratory pressure (Ppeak) (cm H_2_O) (median [IQR]), plateau pressure (cm H_2_O) (mean ± SD), dynamic lung compliance (mL/cm H_2_O) (median [IQR]) values in groups.

Ppeak	T1	T2	T3	T4	T5	T6	T7
VCV	16.5 (15.0–18.2)	21.5 (20.0–23.2)	23.0 (21.7–25.0)	23.5 (22.0–26.2)	24.5 (23.0–27.0)	24.5 (23.0–28.0)	26.0 (23.5–29.0)
PCV	15.5 (14.7–17.0)	18.5 (17.7–20.0)	19.0 (18.0–20.2)	19.5 (18.0–21.0)	20.0 (18.7–21.0)	20.0 (18.7–22.0)	20.0 (18.5–21.5)
PCV-VG	13.0 (11.9–14.0)	14.4 (13.8–16.0)	15.4 (13.9–16.4)	16.0 (14.1–17.0)	16.2 (14.4–17.0)	16.1 (14.4–17.7)	16.0 (14.0–17.9)
*P* (Ppeak)*	<.001*	<.001*	<.001*	<.001*	<.001*	<.001*	<.001*
Pplato	**T1**	**T2**	**T3**	**T4**	**T5**	**T6**	**T7**
VCV	14.2 ± 2.5	18.5 ± 2.3	20.1 ± 2.9	20.1 ± 2.9	21.3 ± 2.9	21.9 ± 3.2	23.1 ± 3.2
PCV	13.7 ± 1.8	17.2 ± 1.7	17.8 ± 1.7	18.4 ± 1.7	18.5 ± 1.7	18.6 ± 1.8	18.7 ± 1.9
PCV-VG	11.8 ± 1.6	13.6 ± 1.9	14.0 ± 1.8	14.4 ± 1.8	14.6 ± 1.7	14.7 ± 1.7	14.5 ± 1.8
*P* (Pplato)**	<.001**	<.001**	<.001**	<.001**	<.001**	<.001**	<.001**
Cdyn	**T1**	**T2**	**T3**	**T4**	**T5**	**T6**	**T7**
VCV	37.1 34.7–40.9	30.0 26.6–32.7	29.0 26.3–31.4	28.6 25.1–31.0	28.0 24.9–30.3	27.0 24.5–29.4	27.0 23.0–29.0
PCV	41.7 38.5–42.3	36.4 33.4–37.5	35.5 32.5–36.3	35.0 32.0–36.0	35.0 31.0–35.7	34.0 31.0–35.0	33.9 31.0–34.9
PCV-VG	42.5 40.0–50.0	38.5 36.0–46.2	37.7 35.0–45.2	37.0 35.0–45.1	36.6 34.0–44.2	37.0 34.9–45.0	37.0 34.5–44.6
*P* (Cdyn)*	<.001*	<.001*	<.001*	<.001*	<.001*	<.001*	<.001*

* Kruskal–Wallis test.

** One-way ANOVA test.

**Figure 4.**
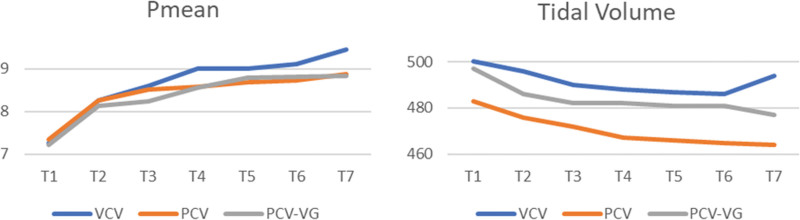
Changes in mean airway pressure (Pmean) (cm H_2_O) and tidal volume (mL) values in the groups.

Pmean increased after transition to pron position in all 3 groups. There was no significant difference in Pmean between the groups in both pron and supine positions (*P* > .05). There was no significant difference in VT levels between the groups (*P* > .05) (Fig. 4).

## 4. Discussion

The aim of this study was to compare PCV, VCV and PCV-VG modes and to determine the optimum ventilation mode for patients undergoing spinal surgery in pron position under general anesthesia. PCV-VG ventilation mode showed lower Ppeak and Pplato values, higher Cdyn and oxygenation values compared to PCV and VCV modes. It was concluded that the use of PCV-VG mode in the prone position may be advantageous for patients.

Prone position is one of the positions with high complication rates. Restriction of chest expansion in the prone position, decreased elasticity of the chest wall, obesity, muscle relaxants and increased intra-abdominal pressure are the most common reasons that decrease respiratory compliance.^[8]^

In their study on positions and respiratory mechanics, Krayer et al^[9]^ reported that the end-expiratory position of the diaphragm did not change after induction of anesthesia and muscle relaxation in the supine position, whereas there was a marked cephalic volume shift in the pron position. The movement patterns of the diaphragm during mechanical ventilation in the supine position are the same, while in the pron position most of the movement is in the dorsal region. In previous studies, as in our study, while dynamic compliance values decreased, Ppeak values increased as expected due to upward movement of the diaphragm in the transition from supine to pron position.^[9]^

Palmon and et al^[10]^ tested the hypothesis that impairment in ventilatory function is related to the patient’s body structure and the surgical frame used to support the patient in the pronated position. In this study, seventy-seven adult patients were divided into 3 groups according to BMI: Normal (n = 36) ≤ 27 kg/m^2^, overweight (n = 21) 28 to 31 kg/m^2^ and obese (n = 20) ≥ 32 kg/m^2^. Patients were placed in the pron position supported by chest rolls, Wilson frame or Jackson spinal surgical table (Jackson table) according to the surgeon’s preference. It was reported that when the patients were turned to the pronated position on the Wilson frame, an increase in Peak pressure and a decrease in pulmonary compliance were observed in each group. It was emphasized that in patients who took pron position on the Wilson frame, an increase in MAP was found in the obese group, while no increase was observed in the overweight or normal groups. In patients who returned to the pronated position on the chest roll, an increase in Peak pressure and a decrease in pulmonary compliance were observed in all groups. Changing to the pronated position on the chest rolls had no effect on MAP in any group. There was no significant change in Peak pressure, compliance or hemodynamic values when changing from supine to pronated position when using the Jackson table, regardless of body structure. In conclusion, this study emphasized that in patients undergoing spinal surgery in the prone position, an increase in Peak pressure and a decrease in lung compliance were observed when moving from the supine to the pronated position when using chest rolls or the Wilson frame, whereas these changes were not observed when using the Jackson table. These devices aim to elevate the anterior surface of the body so that the abdomen can hang freely and the abdominal viscera do not interfere with the inspiratory movement of the diaphragm.^[10]^ Unlike the Jackson table, the Wilson frame and chest rolls are surgical frames that do not allow complete free sag of the abdomen, especially in the overweight and obese population; therefore, these devices may result in increased Peak and decreased respiratory volumes. As a result of this study, it can be said that the Jackson table is the best table to use in spine surgery in the cervical, thoracic and lumbar regions to optimize respiratory mechanics by reducing abdominal and thoracic pressures.^[10]^

Unlike this study, we included patients with a BMI of maximum 30 in our study and we used chest rolls in all patients when changing to pron position. In our study, we determined that there was an increase in Peak pressure and a decrease in compliance values when changing from supine to pron position in all groups. In our study, we aimed to find the ideal ventilation mode while trying to give the optimum position to minimize the negative effects in all patients in whom we used chest rolls.

Soro et al^[11]^ reported a 17% decrease in dynamic compliance after transition from supine to pron position in 14 patients who underwent elective posterior spinal surgery for more than 3 hours. They reported that this was due to the support material they placed under the chest in the pron position during spine surgery that applied pressure on the chest wall.

Wahba et al^[12]^ In their study in which they performed elective lumbar decompression surgery on 20 patients from the ASA I-II group, they reported that the pron position significantly increased functional residual capacity (FRC) and improved oxygenation, whether the patients were underweight or obese. They showed reduced ventilation-perfusion mismatch as the most likely reason for the improvement. They also emphasized that PaCO2 values did not change in their study. The researchers concluded that increased CO2 excretion and decreased EtCO2 value due to increased VT caused by increased elasticity of the chest wall in the pronated position. Wahba et al concluded in their study that the PaCO2-PETCO2 gradient increased after the anesthetized patient was placed in the pron position.^[12]^

In our study, we observed that PaO_2_ values increased in the prone position in all 3 groups. Likewise, unlike the study by Wahba et al^[12]^ in our study, we found that PaCO_2_ values in the pron position decreased in all 3 groups, but there were no significant differences between the groups.

Manikandan et al^[13]^ reported in their study that alveolar dead space decreased in the pron position and increased in the supine position in 21 patients, 17 patients in the lateral position and 31 patients in the pron position. In their blood gas analysis, it was revealed that PaO_2_ increased in all groups after induction. They concluded that PaO_2_ increased from 154 to 166 mm Hg in the lateral position and from 141 to 159 mm Hg in the pron position. They found a statistically insignificant decrease in PaO_2_ values in the group that continued in supine position.^[13]^

Likewise, in our study, we observed that PaO_2_ values increased in all 3 groups in the pronated position. The highest PaO_2_ and peripheral oxygen saturation values were found in the PCV-VG group.

Jun Pu et al^[14]^ reported that PaO_2_ values were better in PCV-VG mode in their study comparing PCV-VG and VCV ventilation modes for one-lung ventilation (OLV) during thoracic surgery. The researchers associated this with the decrease in pulmonary shunt in PCV-VG mode.^[14]^

Tuğrul et al^[15]^ reported that PCV improved arterial oxygenation during OLV compared to VCV in their study including patients with lower forced vital capacity.

However, Unzueta et al^[16]^ stated that they could not support these benefits of PCV mode in their study. They emphasized that this result was related to the fact that maintenance of minute ventilation was not mandatory in PCV.^[15,16]^

In our study, we tried to keep the target VT as 6 to 8 mL/kg in all 3 modes. When we analyzed our data, although the lowest VT was observed in PCV mode at all time points, we did not find a statistically significant difference between the 3 groups in terms of VT.

Melike Korkmaz Toker et al^[17]^ compared PCV-VG ventilation mode to VCV in obese patients undergoing laparoscopic hysterectomy and mean PaO_2_ levels were significantly higher in the PCV-VG group than in the VCV group at every time point after pneumoperitoneum in the trendelenburg position.

In our study, the highest PaO_2_ values were found in the PCV-VG group at all time points.

Hu et al^[18]^ compared the effects of VCV and PCV-VG modes on circulation, pulmonary function and lung injury during OLV. They reported that patients who underwent PCV-VG mode showed lower P-peak levels. They concluded that PCV-VG mode was more advantageous in terms of pulmonary function and lung protection than VCV mode during OLV.

Similarly, Jun Pu et al^[14]^ found that PCV-VG produced better patient oxygenation, significantly lower peak airway pressure, plateau pressure and lower mean airway pressure in their study comparing PCV-VG and VCV ventilation modes for OLV during thoracic surgery. These results suggest that flow-slowing PCV-VG may be superior to VCV in terms of alveolar ventilation and gas distribution.^[19]^ Lin et al^[20]^ obtained significantly lower values in Ppeak and higher values in PaO_2_ in PCV and PCV-VG groups compared to VCV group during OLV in patients undergoing thoracic surgery.^[20]^

In our study as well as in the study by Jun Pu et al^[14]^ and Fei Lin et al^[20]^ the VT was similar in all ventilation modes, indicating that PCV-VG can indeed provide patients with a predetermined ventilation volume. In our study, we can say that PCV-VG mode is more advantageous in terms of pulmonary function than VCV mode in the pron position as well as in patients with one-lung ventilation.

There are also many laparoscopic surgery studies showing lower Ppeak values in PCV and PCV-VG compared to VCV mode.^[21–23]^

Dion et al^[22]^ emphasized that PCV-VG and PCV were superior to VCV ventilation in their ability to provide ventilation with the lowest PIP in adolescents and young adults undergoing laparoscopic bariatric surgery, but they did not detect a difference between oxygenation (PaO_2_), ventilation (PaCO_2_), and hemodynamic variables.^[23]^

In the meta-analysis by Han et al,^[24]^ data from 8 randomized controlled trials including 454 patients who underwent spinal surgery in the pron position were included. As a result of this meta-analysis, it was shown that Ppeak and CVP significantly increased in the VCV mode compared to the PCV mode. It was also emphasized that PCV mode had higher Cdyn and PaO_2_/FiO_2_ values than VCV. However, it was stated that there was no significant difference between PCV and VCV in terms of POB, Hb, HCT, HR and MAP values. In our study period, the PCV-VG group had lower Ppeak and Pplato values than the PCV and VCV group in both supine and prone positions. The highest Ppeak and Pplato values were observed in group VCV. In all 3 groups, a significant decrease in dynamic compliance values was observed in the transition from supine to pronated position. Cdyn was higher in Group PCV-VG than in Group PCV and Group VCV in both supine and pron positions during the study period. The lowest Cdyn was observed in Group VCV. In our study was similar results of Han et. al. study, there is no obvious difference between PCV and VCV in terms of hemodynamics variables such as HR and MAP.

Toker et al^[17]^ reported that PCV-VG ventilation mode limited peak inspiratory pressure, decreased driving pressure and increased dynamic compliance compared to VCV in obese patients undergoing laparoscopic hysterectomy.^[17]^

Fei Lin, et al^[20]^ reported that there was no statistically significant difference in the hemodynamic results of the PCV and PCV-VG groups compared to the VCV group during OLV in patients undergoing thoracic surgery.

In our study, no significant difference was found between the VCV, PCV, PCV-VG groups in the supine and pron position, but a decrease (10–15%) in HR, SAB, DBP, and OAB values was found after the patients were given the pron position in all 3 groups.

One of the limitations of our study is that the effect of ventilation modes on surgical bleeding was not determined. However, since our study included patients who underwent spinal surgery in the prone position and whose bleeding profiles may be different, the amount of bleeding was not included in our study. In future studies, the effects of these ventilation modes on surgical bleeding in cases undergoing spinal surgery in the prone position should be evaluated.

As a result, in this study, lower Ppeak and Pplato values, higher Cdyn and oxygenation values were detected in PCV-VG breathing mode compared to PCV and VCV modes. As a result of this study, it was determined that PCV-VG mode may be more advantageous for patients in pron position, which is one of the challenging positions with high complication rates.

## Author contributions

**Conceptualization:** Ferim Sakize Gunenc, İlkana Seyidova.

**Data curation:** Ferim Sakize Gunenc, İlkana Seyidova, Koray Ur, Volkan Hanci.

**Formal analysis:** Ferim Sakize Gunenc, İlkana Seyidova, Volkan Hanci.

**Methodology:** Ferim Sakize Gunenc, İlkana Seyidova, Sule Ozbilgin, Koray Ur, Volkan Hanci.

**Software:** Volkan Hanci.

**Supervision:** Ferim Sakize Gunenc.

**Writing – original draft:** Ferim Sakize Gunenc, İlkana Seyidova, Sule Ozbilgin, Volkan Hanci.

**Writing – review & editing:** Volkan Hanci.
